# Symbiont-mediated RNA interference in insects

**DOI:** 10.1098/rspb.2016.0042

**Published:** 2016-02-24

**Authors:** Miranda M. A. Whitten, Paul D. Facey, Ricardo Del Sol, Lorena T. Fernández-Martínez, Meirwyn C. Evans, Jacob J. Mitchell, Owen G. Bodger, Paul J. Dyson

**Affiliations:** Institute of Life Science, College of Medicine, Swansea University, Singleton Park, Swansea SA2 8PP, UK

**Keywords:** RNA interference, symbiotic bacteria, biocide, insect, Chagas disease

## Abstract

RNA interference (RNAi) methods for insects are often limited by problems with double-stranded (ds) RNA delivery, which restricts reverse genetics studies and the development of RNAi-based biocides. We therefore delegated to insect symbiotic bacteria the task of: (i) constitutive dsRNA synthesis and (ii) trauma-free delivery. RNaseIII-deficient, dsRNA-expressing bacterial strains were created from the symbionts of two very diverse pest species: a long-lived blood-sucking bug, *Rhodnius prolixus*, and a short-lived globally invasive polyphagous agricultural pest, western flower thrips (*Frankliniella occidentalis*). When ingested, the manipulated bacteria colonized the insects, successfully competed with the wild-type microflora, and sustainably mediated systemic knockdown phenotypes that were horizontally transmissible. This represents a significant advance in the ability to deliver RNAi, potentially to a large range of non-model insects.

## 1. Introduction

Insects and arthropods are of enormous significance as agricultural or stored product pests and vectors of disease, as well as many species being beneficial such as pollinators. The challenge to control pests and protect beneficial species is of increasing concern, as conventional measures using chemical insecticides are being undermined by the evolution of resistance and a moratorium on the use of certain chemicals due to their non-specificity. This underscores why insight into the biology of insects is vital, so that alternative control measures can be devised. Several insect genome projects focused on disease vectors and agricultural pests are currently in progress, and the ambitious i5 k project (5000 insect and other arthropod genome initiative) is now gaining momentum. To exploit these emerging genetic resources, methods to investigate and exploit gene function are urgently required.

The extraordinary insight into animal biology and, in particular, development provided by classical genetic analysis of the model organism *Drosophila melanogaster* has provided a powerful incentive to develop molecular genetic tools to interrogate and manipulate specific gene functions in this insect. These tools are largely dependent on creating transgenic flies by injecting embryos with genetic constructs based on transposable elements such as the P-element that can integrate into the genome. A current goal of the *Drosophila* genome project is to ascertain the function of each gene. One favoured method to obtain specific gene knockdowns in larvae and adults is to create transgenic lines of flies by injecting P-element constructs expressing ‘snap-back’ RNA to evoke RNA interference (RNAi) [1]. However, the reverse genetics techniques developed for *Drosophila* do not translate well to other species, leaving a significant technological gap in our ability to study and manipulate these insects, despite the obvious need to do so.

While RNAi is considered the technique of choice for insect reverse genetics, it is constrained by the means to deliver double-stranded (ds) RNA to insects less genetically tractable than *Drosophila*. Delivery in some species has been demonstrated by injection, with the biological caveats that surviving insects often suffer trauma [2] and upregulated immune responses [3] that could mask or lead to misinterpretations of the phenotype. In addition, injection is labour-intensive and especially challenging for small insects. For larger, long-lived insects, the transient nature of RNAi after injection is also an issue. Ingestion of dsRNA is an alternative as, typically, the invertebrate gut is well adapted for the uptake of dsRNA [4]. Consequently, RNAi can be achieved by either mixing comparatively large amounts of purified dsRNA into food, spiking food with recombinant *Escherichia coli* cells expressing dsRNA, or, for phytophagous insects, generating dsRNA-expressing transgenic plants. These routes are compromised by the transient nature of RNAi evoked by ingested dsRNA (necessitating continuous or repeat exposures) (e.g. [5]), induction of immune reactions in the case of ingestion of sufficient *E. coli* cells, which are not usually present in the gut [6,7], and the time and expense required to generate transgenic plants.

Insect bacterial symbionts play an important role in many aspects of the biology of their hosts. Whereas obligate endosymbionts such as *Buchnera* have typically undergone major genome reduction [8], the guts of different insect species frequently harbour facultative symbionts that can be cultured *in vitro*. One such symbiont is *Rhodococcus rhodnii*, which has evolved to live in the midgut of colonial triatomine insect vectors of Chagas disease such as *Rhodnius prolixus*, being transmitted horizontally from adults to juveniles via coprophagy [9]. These large haematophagous insects have an average life cycle of greater than five months' duration [10]. Aposymbiotic insects not carrying *R. rhodnii* suffer increasingly higher rates of mortality in each stage of development, which has been attributed, in part, to the role of the bacteria in synthesizing B vitamins (e.g. [9,11]). This association has been exploited in paratransgenesis, whereby the bacteria have been genetically manipulated to express trypanocidal proteins (e.g. cecropin A [12]) in the insect gut to interrupt the life cycle of *Trypanosoma cruzi* parasites.

Here, we describe a novel way to exploit the association between culturable symbiotic gut bacteria and their hosts: to constitutively deliver dsRNA to evoke RNAi in the host. The technology provides a feasible means both to investigate gene function and as a biocide to control insect population size. To highlight its broad applicability, we demonstrate the technology using two very different species of symbiont (one, a Gram-positive actinobacterium and the other, a Gram-negative gamma proteobacterium), in two contrasting insect species: the large, long-lived haematophagous insect *R. prolixus* (in which the dsRNA expression cassette is stably integrated into the chromosome of the symbiont, removing the requirement for constant antibiotic selection) and the much smaller, polyphagous *Frankliniella occidentalis* (western flower thrips) that has a short life cycle. The latter species is a major global threat to agriculture that has extended its range from its native North America to all cultivated continents in the past 25 years, concomitantly developing considerable pesticide resistance [13].

## 2. Material and methods

### (a) Insects

*Rhodnius prolixus* (Hemiptera: Reduviidae) were from a long-established captive colony at Swansea University and were reared at 27°C, 80% relative humidity and a 12 L : 12 D cycle, in plastic pots containing filter paper. Defibrinated horse blood (TCS Microbiology, Buckingham, UK) was fed fortnightly to the insects using a membrane feeding system [14]. Western flower thrips, *F. occidentalis* (Thysanoptera: Thripidae), were generously supplied by Koppert Biological Systems (The Netherlands) and maintained on chrysanthemums, pine pollen and runner beans at 70–80% relative humidity, 26–27°C, with a 14 L : 10 D cycle, respectively.

### (b) Bacterial strains

*Rhodococcus rhodnii* LMG5362, isolated from the hindgut of *R. prolixus*, was obtained from the Belgian Co-ordinated Collections of Micro-organisms. *Rhodococcus rhodnii* was routinely cultured in TSB at 28°C. BFo2 was isolated from homogenized surface-sterilized *F. occidentalis* and grown at 30°C in liquid culture (LB, with shaking) and on the surface of LB agar plates. Cloning procedures were performed in *E. coli* JM109 [15] (Promega, Southampton, UK). All recombinant DNA procedures are described in the electronic supplementary material. DNA was introduced into both *R. rhodnii* and BFo2 by electroporation; details of the methods are described in the electronic supplementary material.

### (c) Introduction of recombinant *Rhodococcus rhodnii* to *Rhodnius prolixus*

Recombinant *R. rhodnii* were grown in TSB (+ apramycin 50 µg ml^−1^ and kanamycin 50 µg ml^−1^), washed twice and resuspended in sterile defibrinated horse blood to a previously optimized concentration of 5 × 10^6^ ml^−1^. Insects were then membrane-fed in a class 2 laminar flow cabinet using a 37°C heating platform upon which sterile Petri dishes or smaller tube lids were filled with the blood/bacteria mixture and covered with a sterile Gammex^®^ PF-microthin™ surgical glove (Ansell Healthcare, Brussels) to mimic skin. After feeding to engorgement, the insects were maintained inside plastic containers covered with a gas-permeable sealing membrane (Sigma-Aldrich Co. Ltd, Dorset, UK). Unfed (unengorged) insects were easily identified and could thus be removed. The blood/bacteria mixes used for infection were routinely cultured on TSA (+ antibiotics) after feeding to verify the continuing viability of the bacteria. Aposymbiotic and non-sterile insects of different ages were infected. Aposymbiotic *R. prolixus* were raised in sterile conditions from eggs sterilized for 10 min in 2.5% iodine 2.5% (w/v), 2.5% potassium iodide BP (w/v) and 89% ethanol (v/v), and rinsed twice with autoclaved water.

Lysozyme and phenoloxidase activities, key insect innate immune responses (e.g. [16]) were assessed in *R. prolixus* following ingestion of recombinant *R. rhodnii* to determine whether ingestion of recombinant *R. rhodnii* triggers a systemic immune response. Third to fifth instar insects were either fed with *R. rhodnii* ME315 expressing ds*dagA*, or left unchallenged (controls). Haemolymph collected from a severed limb 48 h after challenge was assayed for lysozyme and phenoloxidase activity using the methods of Dubovskiy *et al*. [17].

### (d) Introduction of recombinant BFo2α to *Frankliniella occidentalis*

Overnight cultures of recombinant BFo2α containing the dsRNA expression plasmids were pelleted by centrifugation (3000*g* for 2 min) and washed by resuspension in LB, then resuspended to 5 × 10^6^ ml^−1^ in a previously optimized artificial feeding mixture (20% (v/v) LB, 2.4% (w/v) sucrose, 0.32% (w/v) NaCl and 0.03% (w/v) methylene blue). *Frankliniella occidentalis* thrips of all developmental stages were membrane-fed on the feeding mixture as the only food source for 2–4 days from an inverted Bijou bottle reservoir covered by stretched Parafilm^®^. Methylene blue was included to non-invasively identify which insects had fed (the blue colour in the gut being visible through the cuticle under low-power magnification). Bacterial growth and viability in the feeding mixture was confirmed at the beginning and end of each experiment by culturing on LB agar supplemented with 1.5% (w/v) sucrose and the appropriate selective antibiotic (apramycin for BFo2*α* expressing ds*dagA*; apramycin and ampicillin for BFo2*α* expressing ds*Tub*). The gut contents of randomly selected, surface-sterilized thrips were also cultured on selective media to verify the viability and population of ingested BFo2α strains. Additional controls involved using overnight cultures of ds*Tub*-expressing BFo2α, which were heat-killed by incubation at 65°C for 20 min prior to incorporation into feeding mixtures as above. For sorting, thrips were anaesthetized with CO_2_ via a Flystuff Flypad (Genesee Scientific, San Diego, CA, USA).

### (e) Phenotypic and molecular assessments of RNA interference

Details for how gene knockdown phenotypes were assessed, validation of mRNA abundance by qRT-PCR and statistical analyses of these data are described in the electronic supplementary material.

## 3. Results

### (a) *Rhodnius prolixus*: bacterial introduction, transmission and fitness effects

**Figure 1.**
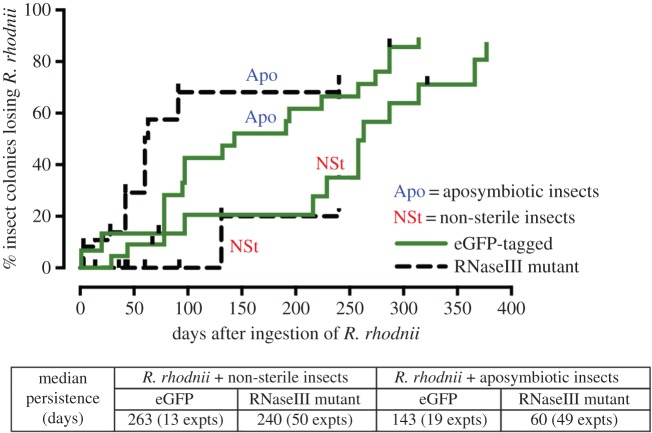
Persistence of eGFP-expressing and RNaseIII mutant *R. rhodnii* in *R*. *prolixus*, after a single infective feed*.* The survival chart compares the persistence rate of *R. rhodnii* following a single *per os* exposure in non-sterile and aposymbiotic insects. Presence of the bacteria was defined as more than 25 CFUs in samples of excreta, as a proxy for digestive tract colonization. The vertical axis indicates the cumulative percentage of insects losing introduced *R. rhodnii* (assessed over a period of 240 days for RNaseIII mutant ME315 strain *R. rhodnii* and 377 days for eGFP-tagged *R. rhodnii*, commencing 24 h after infective feeding). Vertical ticks indicate censored data (i.e. mortality events in *R. rhodnii* infected insects). Median bacterial persistence times are indicated in the matrix (with the number of repeat experiments indicated in parentheses). While persistence was compromised in bacteria fed to aposymbiotic insects (*p* < 0.05 compared with non-sterile insects), there was no significant difference between non-sterile insects fed eGFP-tagged bacteria versus RNaseIII mutant bacteria (Mantel–Cox log-rank test).

To investigate parameters concerning introduction of recombinant *R. rhodnii* and their transmission between individual *R. prolixus*, plus associated fitness effects, we first conducted experiments with *R. rhodnii* expressing *eGFP* but not dsRNA, and later with *R. rhodnii* ME315 expressing control ds*dagA* (dsRNA against a bacterial agarase gene, used as an off-target control for subsequent experiments). These bacteria were mixed with sterile blood and introduced to aposymbiotic first-instar nymphs and symbiont-containing insects (i.e. harbouring their endogenous gut microflora) at different developmental stages by membrane feeding. The inoculation of insects by the recombinant bacteria was monitored by culture of gut contents on selective media, followed by PCR of the resulting bacterial colonies. Insects were equally likely to feed on a blood meal spiked with bacteria as they were on sterile blood. Recombinant *R. rhodnii* were sufficiently robust to compete with the existing microflora of normal, symbiont-containing insects of all ages. The bacteria were detectable in the insect midgut, faeces and occasionally hindgut within 48 h of ingestion (figure 1). Moreover, these bacteria persisted in the insects for more than 250 days after their initial uptake, having no detectable impact on insect fitness. Median persistence times following a single *per os* exposure to the bacteria were 263 days for non-sterile insects with eGFP-tagged bacteria and 240 days for non-sterile insects with RNaseIII mutant strain ME315 *R. rhodnii*, but significantly less for aposymbiotic insects exposed to the recombinant bacteria (figure 1).

EGFP-tagged *R. rhodnii* were not detected in the haemolymph of fed insects, nor was there evidence of immune activation or systemic septic shock as measured by phenoloxidase and lysozyme activity (see electronic supplementary material, figure S1, for data and details), indicating bacterial confinement to the gut. Aposymbiotic insects populated with recombinant *R. rhodnii* developed faster and reached sexual maturity earlier than non-exposed controls, and furthermore ingestion of modified bacteria by aposymbiotic insects rescued the incidence of moult deformities leading to premature mortality. We measured a 53% mortality rate over the first eight weeks of life of aposymbiotic insects, compared to 24% mortality of wild-type insects and 22% mortality of aposymbiotic insects infected with EGFP-tagged *R. rhodnii*, indicating the beneficial symbiotic characteristics of the bacteria are retained.

By removing eggs or first-instar hatchlings to sterile rearing chambers, we could assess vertical transmission from parents fed with recombinant bacteria. Normal and surface-sterilized eggs were also compared. Vertical transmission of eGFP-expressing *R. rhodnii*, however, was negligible (see electronic supplementary material, table S1). Horizontal (environmental) transmission was frequent in older nymphs (i.e. second-instar and older), provided they were exposed to, and fed in, the presence of contaminated faeces (table 1). Environmental acquisition of modified bacteria was efficient and stable. Thus the recombinant bacteria have the potential to spread efficiently through a colony of insects, and disruption of RNaseIII function appears to have little effect on symbiont fitness.

**Table 1.** Evidence for horizontal (environmental) transmission of eGFP-expressing or RNaseIII mutant *R. rhodnii* in *R. prolixus*.

% of recipient insects in which horizontal transmission of modified *R. rhodnii* was detected	
combinations of recipient insects older than first-instar + donor insects older than first-instar carrying eGFP-tagged *R. rhodnii* (*n* = 29)	79.3%
combinations of recipient insects older than first-instar + donor insects older than first-instar carrying RNaseIII mutant *R. rhodnii* (*n* = 56)	83.9%
recipient first-instar nymphs + donor insects older than first-instar carrying RNaseIII mutant *R. rhodnii* (*n* = 16)	25% (*p* < 0.001)
recipient first-instar nymphs + donor insects older than first-instar carrying eGFP-tagged *R. rhodnii* (*n* = 33)	0% (*p* < 0.001)
recipient first-instars hatched in the presence of adults carrying eGFP-tagged *R. rhodnii* but fed in isolation (*n* = 36)	0% (*p* < 0.001)

### (b) Assessing bacterial symbiont-mediated RNAi in *Rhodnius prolixus*

Systemic RNAi in *R. prolixus*, usually via introduction of synthetic dsRNA by injection, has been demonstrated for a few genes and notably those encoding the ferric haem nitrophorin proteins expressed in salivary glands [18]. Knockdowns present an obvious phenotype after their next feed in that the normal cherry-red coloured salivary glands appear pale or colourless. For preliminary experiments, assessing efficacy of symbiont-mediated RNAi, we therefore introduced dsRNA expression cassettes for *Nitrophorin-1* (ds*NP1*), *Nitrophorin-2* (ds*NP2*) or an off-target control (ds*dagA* for agarase) into the chromosome of both wild-type *R. rhodnii* and an RNaseIII-deficient mutant, strain ME315. The mutant was created to circumvent the rapid endogenous degradation of RNA, which we established prejudices the stable synthesis of dsRNA.

Aposymbiotic second- and third-instar nymphs were populated during a blood meal with *R. rhodnii* ME315 strains expressing ds*NP1*, ds*NP2* or ds*dagA*. The insects were subsequently sacrificed after a further blood meal (eight weeks after initial infection), and their salivary glands dissected. Control (ds*dagA*) glands were cherry-red indicating normal synthesis of salivary nitrophorin proteins. Knockdown of NP1 or NP2 expression resulted in colourless glands, as we also observed after injection of dsRNA (electronic supplementary material, figure S2). In extreme cases of symbiont-mediated NP2 knockdown and double-knockdown of NP1 and NP2, due to combined infection with two symbiont strains, we observed severe tissue wastage and crystallization of the luminal contents (electronic supplementary material, figure S2b,c). A large proportion (more than 60%) of non-sacrificed knockdown insects populated with the ds*NP2* bacteria also exhibited aberrant/abortive feeding behaviours that eventually led to their mortality, likely due to salivary gland dysfunction. In parallel experiments, we populated aposymbiotic nymphs with the wild-type *R. rhodnii* strain containing the dsRNA expression cassettes. No significant knockdown phenotypes were observed. Moreover, dsRNA production could only be detected in the RNaseIII mutant (see electronic supplementary material, figure S3), indicating that stable dsRNA synthesis by the bacteria is a prerequisite for effective RNAi.

### (c) An RNAi-based strategy to control fecundity of *Rhodnius prolixus*

**Figure 2.**
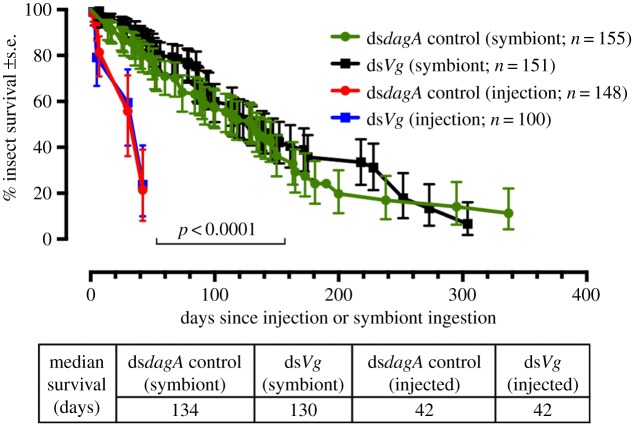
A comparison of the survival of *R*. *prolixus* using two different dsRNA delivery vehicles. The traditional dsRNA injection method was compared with symbiont-mediated RNAi. Fifth instar insects were either micro-injected with 2 µg dsRNA or fed dsRNA-expressing RNaseIII mutant ME315 *R. rhodnii* symbionts. Treatments either targeted the *Vitellogenin* gene (*Vg*, which has a non-lethal phenotype) or the control gene *dagA*. Symbiont-mediated dsRNA delivery incurred a significantly lower mortality compared with injection (*p* < 0.0001, Mantel–Cox log-rank test). In these experiments, the injected fifth instar insects failed to moult to the adult stage.

We focused on gametogenesis, and in particular oogenesis, as a potential biocide target in *R. prolixus* and to demonstrate the capability of the technique to deliver a prolonged horizontally transmissible RNAi. In most insects, a single phospholipoglycoprotein, vitellin (Vt), is the main component of eggs. Indeed, about 80% of the total protein content of *Rhodnius* oocytes comprises Vt [19]. Vt is derived from vitellogenin (Vg), which most insects synthesize in the fat body [20]. We introduced a dsRNA expression cassette for *Vg* into the chromosome of the *R. rhodnii* RNaseIII mutant strain ME315. These recombinant bacteria, or *R. rhodnii* ME315 expressing the off-target control ds*dagA*, were introduced into fifth instar (final juvenile stage) non-sterile insects via two successive blood feeds spiked with the bacteria. For the next 200 days, all subsequent feeds were with sterile blood to demonstrate the longevity of the symbiont-mediated RNAi effect. Post-feeding insect mortality was significantly lower than mortality associated with dsRNA injection and not significantly different between the off-target control symbiont-containing insects and the symbiont-containing insects populated by recombinant bacteria expressing ds*Vg* (figure 2).

Ingestion of ds*Vg*-expressing *R. rhodnii* had a negative impact on *R. prolixus* fecundity. This corresponded to a significant (72.3%) reduction in the overall number of eclosed first-instar offspring per adult female per day, compared with the control group (*p* = 0.018; table 2). The reduction in offspring may be attributed to decreased oviposition, reduced eclosion or a combination of both. The eclosion rate was reduced by a mean of 61.5% (i.e. more time elapsed between egg-laying and hatching) in the ds*Vg*-exposed insects compared with the controls, however this change was not significant (*p* = 0.073; table 2). Oviposition was reduced by 58% in the ds*Vg*-exposed insects, but again not significantly (*p* = 0.111; table 2). Insect death rates and moulting were unaffected (table 2). The observations that fewer eggs appear to have been laid, and fewer eggs appear to have hatched, leading to a significant reduction of eclosed first-instar offspring, suggest an accumulative effect on fecundity whereby some oviposited eggs were infertile, while others were unable to support embryonic development to eclosion.

**Table 2.** Fecundity and survival phenotypes in *R. prolixus* associated with ingested recombinant *R. rhodnii* expressing ds*Vg*.

phenotype	% change compared with controls	*p*-value
adult male mortality	0.0%	0.984
adult female mortality	−23.8%	0.697
larval offspring mortality	−59.8%	0.098
moulting of offspring larvae (overall)	+28.9%	0.509
oviposition (egg-laying)	−58.6%	0.110
emergence of first-instar offspring	−72.3%	0.018
rate of egg eclosion (including eggs that failed to hatch)	−61.5% (i.e. 2.60-fold slower)	0.073

**Figure 3.**
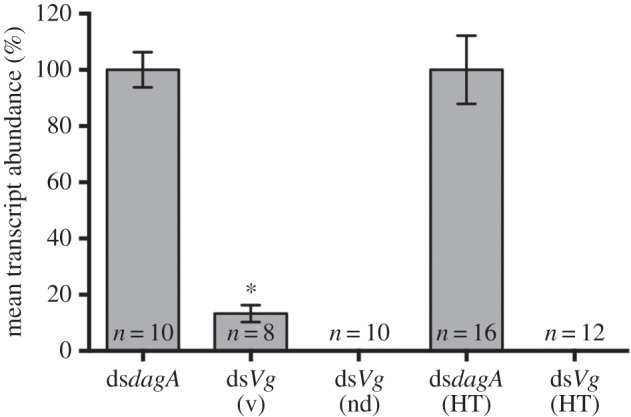
qRT-PCR validation of symbiont-mediated RNAi of vitellogenin. Relative *Vitellogenin* expression in *R. prolixus* individuals normalized to the endogenous control (18S rRNA) was analysed by qRT-PCR in sacrificed adult insects previously infected as fifth instars with recombinant bacteria. These bacteria expressed either ds*dagA* (controls) or ds*Vg*. The bars represent the mean relative *Vg* transcript abundance proportionate to the average relative abundance of *Vg* mRNA in animals treated with ds*dagA* and indicate that symbiont-mediated delivery of ds*Vg* resulted in a significant reduction in vitellogenin expression. In eight insects harbouring bacteria expressing ds*Vg* with detectable, but variable, transcript levels (labelled v), significantly reduced levels (*p* < 0.05, indicated as *) of *Vg* transcript were detected. In 10 insects, no detectable (nd) transcript levels were observed. *Vitellogenin* expression was also measured in individual insects that, as fifth instars, had obtained bacteria by horizontal transfer (HT) either expressing ds*dagA* (*n* = 16) or ds*Vg* (*n* = 12). No *Vg* transcript could be detected in any of the latter group of insects.

To correlate the reduced fecundity phenotype with specific RNAi, qRT-PCR was employed. In the ds*Vg* knockdown insects (*n* = 18), 60% had no detectable Vg transcript (within 50 amplification cycles), while the remaining 40% exhibited variable expression ranging from 4 to 63% of wild-type transcript abundance (figure 3). This correlates with the finding that 60% of these insects (and 67% of the dsAg control insects) supported high gut populations of the recombinants at the time of dissection (as determined by more than 1000 CFUs per dissected gut after growth on selective media), whereas the remaining insects had lower levels of recombinant bacteria (between 83 and 674 CFUs per gut). Within the same samples, amplification of the endogenous control (18S rRNA) was consistent with the control samples (*n* = 10). Control insects fed with recombinants expressing ds*dagA*, in contrast, had normal transcript abundance for *Vg* (*n* = 10) and normal fecundity.

To assess the potential for horizontal (coprophagic) transmission of the fecundity impairment phenotype, fifth-instar symbiont-containing insects were placed in pots containing faeces of insects previously populated with ds*Vg* or ds*dagA* expressing bacteria. Once moulted to adults, the fecundity of the new occupants was assessed over nine weeks, with a complete deficit of oviposition in the ds*Vg* group (*n* = 12), compared with 0.07 eggs per female per day in the controls (*n* = 16). The loss of egg production in all members of this ds*Vg* group correlated with high populations of the recombinant bacteria in the gut of each insect, suggesting that the recombinant bacteria can establish themselves more efficiently when acquired via ‘natural’ coprophagy in comparison to when they are acquired artificially in a blood feed. The Vg transcript could not be detected by qRT-PCR of mRNA isolated from these individuals, in contrast to the 18S rRNA endogenous control that was present at levels similar to those in control samples (figure 3).

### (d) Symbiont-mediated RNAi in *Frankliniella occidentalis*

**Figure 4.**
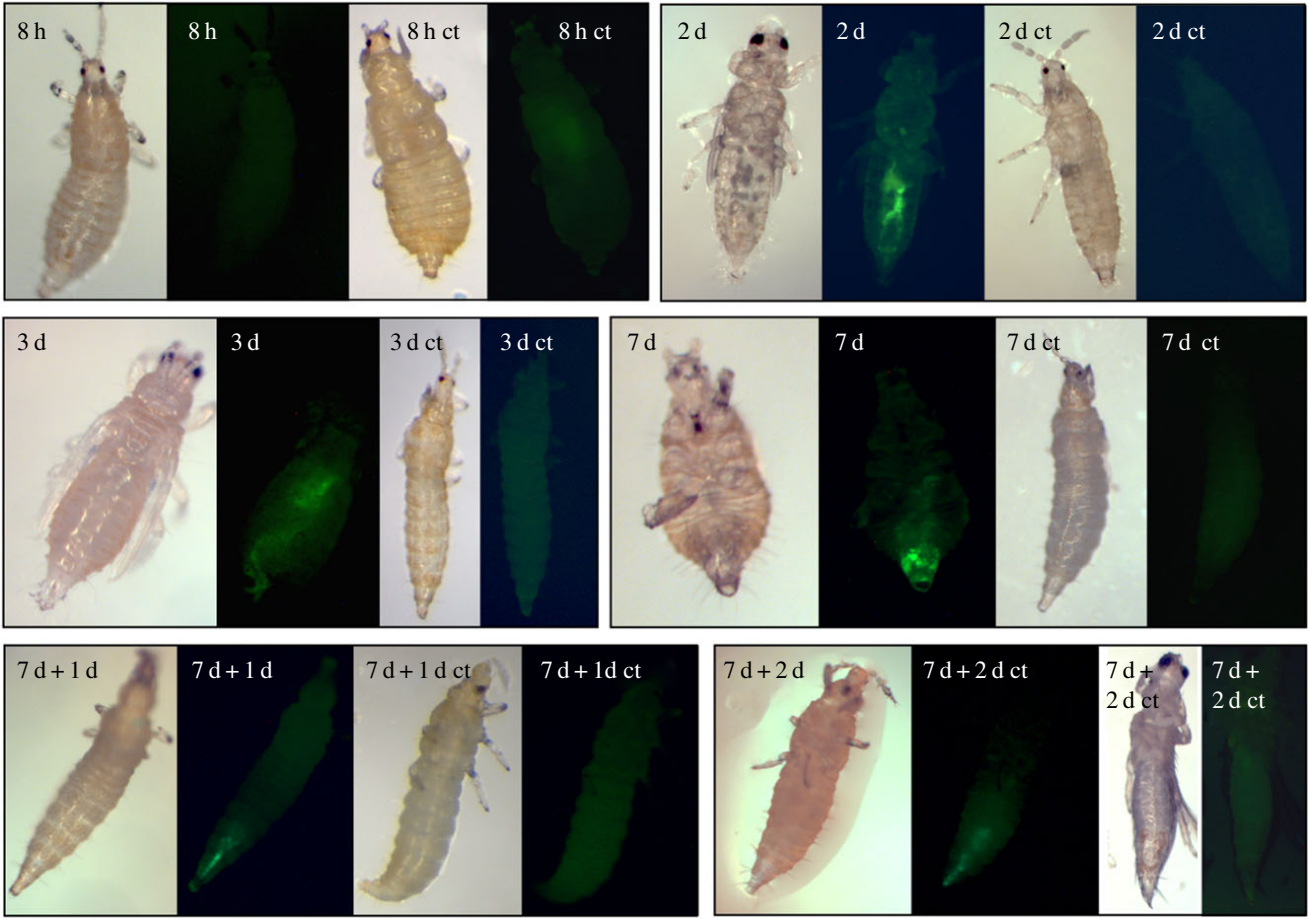
Localization and persistence of BFo2 in the gut of *F. occidentalis* larvae. BFo2 expressing eGFP were fed to first- and second-instar thrips larvae using an artificial feeding system. Green fluorescence was monitored in the gut through the intact cuticle of live insects at regular intervals during dietary exposure. In contrast to dissected tissues, autofluorescence of the gut or other tissues could not be detected through intact cuticle of non-inoculated control insects (indicated as ‘ct’ in the panel). The fluorescent BFo2 could not be detected as early as 8 h, however by 2 days regions of the hindgut and posterior midgut fluoresced strongly, indicating that the recombinant BFo2 bacteria multiplied in the gut before reaching a population that was detectable by fluorescence microscopy. Withdrawal of eGFP-expressing BFo2 from the diet after 7 days did not result in diminished fluorescence in the gut at either 1 day or 2 days (indicated as 7 d + 1 d and 7 d + 2 d) after withdrawal, indicating that the bacteria can persist and compete with wild-type gut bacteria.

We isolated two Gram-negative facultative symbiotic bacterial species from *F. occidentalis*, as has been previously described [21]. One species, called BFo1 (an *Erwinia* species) proved less amenable to genetic manipulation. The second, BFo2 (an Enterobacteriales species that does not group closely with any currently known bacterial species [22]) was consequently used to deliver RNAi. In initial studies, BFo2 was manipulated to express eGFP. This strain was used to optimize experimental conditions for bacterial infection of *F. occidentalis* and to monitor the presence and stability of the bacteria in the guts of infected insects, confirming that recombinant BFo2 can persist and compete with wild-type gut bacteria (summarized in figure 4 and Material and methods).

**Figure 5.**
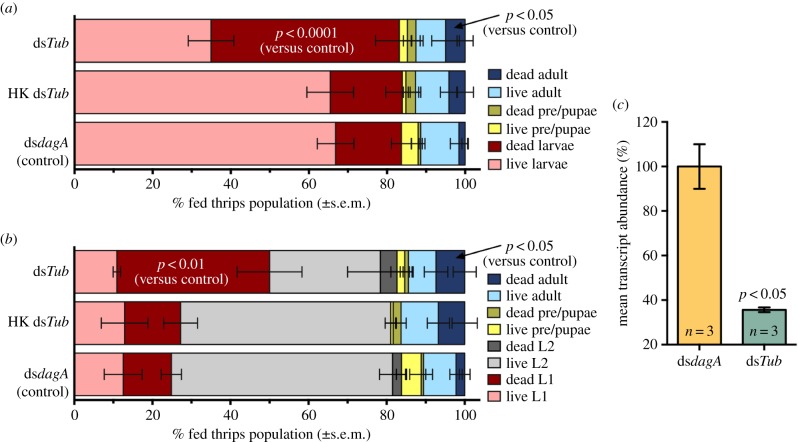
Mortality phenotype of symbiont-mediated *Tubulin* (*Tub*) knockdown and qRT-PCR validation in western flower thrips, *F. occidentalis*. (*a*) Composition of thrips *F. occidentalis* populations following 4 days' oral exposure to modified BFo2α strains expressing *dagA* (control) or ds*Tub*, showing a significant mortality phenotype in the larval and adult stages exposed to ds*Tub*. Data compiled from seven independent experiments; number of fed insects = 234 (control), 150 (heat-killed [HK] ds*Tub*) and 220 (ds*Tub*). Note: the pre-pupal and pupal stages of thrips are non-feeding. (*b*) Additional experiments identifying the first larval stage as most susceptible to the mortality phenotype. Data compiled from four further independent experiments; number of fed insects = 134 (control), 95 (HK ds*Tub*) and 81 (ds*Tub*). Exposure to live BFo2α expressing ds*Tub* significantly increased mortality in adult, larval and particularly L1-stage larval insects (assessed by Fisher's exact test). This phenotype was, however, abrogated when heat-killed bacteria were used. (*c*) Relative *Tub* expression in *F. occidentalis* was normalized to the endogenous control (18S rRNA), analysed by qRT-PCR in whole insects sacrificed at the end of a 48-h period during which they ingested RNaseIII mutant BFo2 bacteria. These bacteria expressed either ds*dagA* (controls) or ds*Tub*. The bars represent the mean relative *Tub* transcript abundance proportionate to the average relative abundance of *Tub* mRNA in animals treated with ds*dag*, and indicate that symbiont-mediated delivery of ds*Tub* resulted in a significant reduction in tubulin expression. Standard deviations represent deviations about the mean of both biological and technical replicates. Each *n* is replicated three times (biological replicates) and represents a pool of approximately 25 insects per treatment per replicate.

To deliver dsRNA, an RNaseIII-deficient mutant, BFo2α, was obtained into which plasmids, directing expression of dsRNA, were introduced. To demonstrate symbiont-mediated gene silencing, we targeted alpha-tubulin (Tub) production that could be essential. Stable bacterial expression of ds*Tub* and the off-target control ds*dagA*, was confirmed by qRT-PCR prior to introducing the bacteria into insects of different ages via an artificial sucrose-based feeding solution. After 4 days, a highly significant mortality phenotype was observed among larvae exposed to ds*Tub*, particularly in the first (L1) larval stage (figure 5; *p* < 0.0001). A small but significant mortality was also observed among the adult *F. occidentalis* (*p* < 0.05). In additional controls, BFo2α expressing ds*Tub* were heat-killed and incorporated into separate feeding mixtures. Heat-killed bacteria failed to elicit the mortality phenotype, highlighting the importance of reintroducing live dsRNA-expressing bacteria as opposed to pre-synthesized dsRNA.

To verify that the observed mortality is correlated with specific RNAi, juvenile thrips (L1 and L2 stages) were sampled 48 h after infection with recombinant BFo2*α*, mRNA extracted and levels of *tubulin alpha1* mRNA quantified. Each experiment revealed depletion of *tubulin* mRNA abundance in infected larval groups (*p* < 0.05; figure 5*c*). Variance in and between experiments is likely due to variable levels of infection between insects.

We also tested whether we could quantify protection to plants afforded by RNAi. Groups of cucumber seedlings were exposed to larval and adult *F. occidentalis* that had been orally infected with BFo2α expressing ds*dagA* (control) or ds*Tub* (*Tubulin* knockdown). After 5 days, the percentage of the leaf surface that was covered with lesions was assessed by image analysis software. Significantly less damage occurred on plants exposed to *F. occidentalis* receiving the ds*Tub* knockdown compared with the controls (*p* = 0.027; electronic supplementary material, figure S4).

## 4. Discussion

We demonstrate symbiont-mediated RNAi in two insect species: one a tropical disease vector, the second a globally invasive agricultural pest. Using synthetic biology, we engineered two dsRNA expression cassettes suitable for diverse applications in a Gram-positive actinobacterium and a Gram-negative gamma proteobacterium that are symbionts of the haematophagous *R. prolixus* and the phytophagous *F. occidentalis*, respectively. These cassettes are designed for insertion of sequences to target expression of any gene via RNAi. Introduction of these cassettes into bacteria deficient in synthesis of RNaseIII permits stable synthesis of specific dsRNA molecules that can be absorbed by the insect gut and induce systemic RNAi, presumably after bacterial cell death and lysis *in insecta*.

An experiment describing RNAi in *R. prolixus* due to expression of a long hairpin dsRNA in *R. rhodnii* was reported during preparation of this manuscript [23]. The description of how this was achieved is incomplete (a single reference to a book published in 1997) with no description of the vector used, no details for how the genetic construct was made and the promoter used to drive expression. Moreover, as we report here, dsRNA is unstable in wild-type *R. rhodnii*, necessitating construction of an RNaseIII mutant. As there is no mention of deriving such a mutant, we are led to question the validity of their claim of symbiont-mediated RNAi. Focusing on substantive differences in experimental design that are explained, their report includes data for short-term RNAi, with constant inclusion of kanamycin antibiotic in blood feeds, suggesting that a non-integrated plasmid has been employed. For our experimental approach, in view of the long *R. prolixus* life cycle and the potential for horizontal (coprophagic) transmission of symbionts between individuals in a colony, we stably integrated the dsRNA expression cassette within the *R. rhodnii* chromosome which removes the requirement for constant antibiotic selection and consequent impact of oral antibiotic ingestion on the physiology and microflora of the insect. Insects at different developmental stages could be sustainably populated with the recombinant bacteria either by feeding on blood mixed with the bacteria or via coprophagy. This is a relatively facile strategy to obtain insect lines in which expression of specific genes is knocked down via systemic RNAi without inducing the trauma associated with injection of dsRNA. Moreover, the continual expression of dsRNA in the gut allows for sustained RNAi so that phenotypic changes related to loss of gene function at each developmental stage can be assessed. We further demonstrate that targeting gametogenesis can significantly reduce fecundity while permitting, due to the long life cycle of the insect, good opportunity for the horizontal transmission of recombinant bacteria, and hence the spread of the impaired-fecundity phenotype within a colony. We also show that it is not necessary to eliminate competition from wild-type gut bacteria, since aposymbiotic insects of either species are not required for successful gut colonization by recombinant bacteria and mediation of the knockdown effect. In fact, the reintroduced bacteria were more persistent in *R. prolixus* containing a normal gut microflora than in aposymbionts, suggesting that there may be beneficial interactions between symbionts and the various microflora of the host's gut. In addition, the persistence of the symbiont may reflect differences between host immune system in the two insect groups. These data imply that a single application of recombinant symbiotic bacteria to a colony of *R. prolixus* could be sufficient to dramatically reduce the colony's viability and consequently reduce transmission of *T. cruzi* in the vicinity. In other experiments, we have demonstrated the viability of the recombinant *R. rhodnii* in insect faeces over several months, with the consequence that insects repopulating an extinct colony can subsequently acquire the impaired-fecundity phenotype.

*Frankliniella occidentalis* is a polyphagous insect that inflicts large losses on a wide range of crops through its feeding and egg-laying behaviours, and also as a vector for plant tospoviruses. It has developed resistance to conventional chemical pesticides. Here, we show that symbiont-mediated RNAi of an essential tubulin gene results in high mortality rates of first-instar larvae within 4 days of infection. As a consequence, this novel biocide offered some protection to the plants used in this study. As the insects did not achieve pupation or subsequent adulthood, transmission of tospoviruses between plants by flying insects could be abrogated. To exploit the emerging genome sequence (www.hgsc.bcm.edu/western-flower-thrips-genome-project), this new technology also offers a powerful means to interrogate gene function. In addition, symbiont-mediated RNAi of an essential insect gene offers biocontrol in a highly targeted fashion, achieved via a combination of the symbiont's host specificity, and the specific sequence of target gene(s). If delivery of the bacteria can be optimized for agricultural applications, it is likely that, in contrast to broad-range chemical and biological control agents, symbiont-mediated RNAi can be employed to control populations of specific insect species while not affecting beneficial species such as pollinators. Specificity of this biocide is afforded by two factors: firstly, available evidence indicates that the host range of facultative bacterial insect symbionts is very limited, due to coevolution of the host and its symbiont, the latter typically undergoing genome reduction as an adaptation to its host environment [24], and secondly, a sequence targeted via RNAi can be selected that is relatively specific to a given insect pest. In addition, any gene can potentially be targeted, or multiple genes to attack all stages of the insect's life cycle. Exploiting this versatility can prevent the emergence of resistance to this novel technology.

It is expected that symbiont-mediated RNAi would be effective in other insect species. The unifying prerequisite is that the insects harbour culturable symbionts, a criterion already known to be met by many globally important insect species such as *Aedes* and *Anopheles* mosquitoes [25,26], tsetse flies [27], whitefly [28] and honeybees [29]. Some insect species are considered refractory to systemic RNAi following ingestion of dsRNA molecules, possibly due to nuclease activity in the midgut digestive juices [30]. Symbiont-mediated RNAi may offer more chance for success in these species, by virtue of continuous and potent synthesis of dsRNA achieved through careful choice of a sufficiently strong promoter. Furthermore, it may be possible to use symbionts that inhabit a gut region that is distant from the focus of nuclease activity. BFo2, for example, localize around the anterior hindgut, close to the origin of the Malpighian tubules [31] (figure 4). In other insects, such as *Drosophila*, a lack of systemic RNAi may be due to the absence of the molecular machinery for cell–cell transmission of dsRNA, as reviewed in [32].

In conclusion, symbiont-mediated RNAi is a powerful means to interrogate gene function in invertebrates. We have demonstrated this transformative technology in two evolutionarily distinct insect species. Based on this success, we believe the technology can potentially be translated to address gene function in many diverse arthropod species, dependent on exploiting facultative gut symbiotic bacteria specific to these species. In addition, the technology provides a potential means of highly targeted biocontrol against tropical disease vectors and agricultural pest species.

## Supplementary Material

Supplementary Material

## Data accessibility

The datasets supporting this article have been uploaded as part of the electronic supplementary material. In addition, raw data files for qRT-PCR experiments are available at http://dx.doi.org/10.5061/dryad.sf937. Genetic constructs can be accessed by contacting the authors.
