# Supplementary material for: Symbiont-mediated RNA interference in insects
Source: Proc Biol Sci. 2016 Feb 24;283(1825):20160042. doi: 10.1098/rspb.2016.0042 (PMC4810840; doi:10.1098/rspb.2016.0042)
Supplement: Supplementary Material [file rspb20160042supp1.pdf]

SUPPLEMENTARY MATERIAL FOR THE PAPER:

Symbiont-mediated RNA interference in insects

Miranda M. A. Whitten, Paul D. Facey, Ricardo Del Sol, Lorena T. Fernández-Martínez, Meirwyn C. Evans, Jacob J. Mitchell, Owen G. Bodger & Paul J. Dyson.

SUPPLEMENTRAY METHODS

Strains and plasmids:

Strains	genotype/comments	Reference
E. coli JM109	F'*traD36 proA*$^+$*B*$^+$*lacIq*Δ(*lacZ)M15*/Δ (*lac-proAB) glnV44 e14- gyrA96 recA1relA1endA1 thi hsdR17*	Yanisch-Perron et al., 1985 [1]
**Plasmids**		
pIJ2925	bla, *lacZ*	Kieser et al., 2000 [2]
pRNA1	pIJ2925 containing ~759bp BFo2 r*naseIII* PCR product	This study
pRNA2	pIJ2925 containing apramycin disrupted BFo2 *rnaseIII* PCR product	This study
pIJ790	λ-RED (gam, bet, exo), cat, araC, rep101ts	Gust et al., 2003 [3]
pQM5062	pMOD+*Tn5062*, Ampicillin^R and Apramycin^R	Bishop et al., 2004 [4]
pFP16	pIJ8600 containing *eGFP* fused to a thiostrepton inducible promoter, Apramycin^R	This study and Sun et al., 1999 [5]
pLL10	Two T7 promoters in reverse orientation, Ampicillin^R	Blandin et al., 2002 [6]
pMW101	dsRNA expression cassette, two convergent *dapA* promoters flanking *dagA*	This study
pIJ8600	*ϕ*C31 *int* and *attP* functions, Apramycin^R	Sun et al., 1999 [5]
pMW43	*R. rhodnii rnaseIII* 2kb fragment ligated into pUC18; Ampicillin^R	This study
pMW44	pMW43 containing Kanamycin^R cassette inserted at *MluI* site of *R. rhodnii rnaseIII* fragment. Ampicillin^R and Kanamycin^R	This study
pMW102	pIJ8600 containing pMW101 with *dagA* flanked by NdeI, Apramycin^R	This study
pMW103	pMW102, EcoRV-excised pCR2.1. Destination plasmid for *R. prolixus* gene fragments, Apramycin^R	This study
pME6	Ampicillin^R and Kanamycin^R	Fernández-Martínez et al., 2009 [7]
pEX-A	Ampicillin^R, *lac* promoter	Eurofins MWG Operon (Ebersberg, Germany)

Electroporation method for *R. rhodnii*:

Plasmids were introduced into *R. rhodnii* cells in mid-log phase growth by electroporation using a MicroPulser (BioRad, Hemel Hempstead, UK). To obtain electrocompetent cells, *R. rhodnii* were cultured in TSB, washed twice with ice-cold 10% glycerol, and concentrated 10-fold in ice-cold 10% glycerol. Immediately before electroporation, competent cells were mixed with DNA (final concentration 0.1 – 1.0 µg/ml). The electroporation was performed in one 1.8 kV pulse in electrocuvettes with gaps of 1 mm. Pulsed cells were immediately diluted 5-fold with TSB and recovered at 28°C for 24 hours, then plated on TSA with 50 µg/ml antibiotic (either kanamycin for RNaseIII mutants, or apramycin for introduction of the expression cassette). The backbone of the expression cassette plasmid is a pIJ8600 derivative vector that is able to integrate into the ΦC31 *attB* chromosomal site of *R. rhodnii.* Consequently, the expression cassette is maintained stably without recourse to constant antibiotic selection.

Recombinant DNA procedures

(1) Production of eGFP-expressing *R. rhodnii* and BFo2
pFP16 is an apramycin-resistant derivative of pIJ8600 that contains eEGFP fused to a thiostrepton-inducible promoter. pFP16 was introduced by electroporation. EGFP-expressing *R. rhodnii* were confirmed by microscopy and PCR (see below for primers). To create constitutively eGFP-expressing BFo2, the eGFP gene was inserted into a 138 bp synthetic expression cassette, which is described below.

(2) *G*ene fragments and *in vitro* dsRNA synthesis
Fragments of *R. prolixus* genes encoding vitellogenin (Vg), nitrophorin-1 (NP1) and nitrophorin-2 (NP2) were obtained by PCR amplification using (respectively) primers RP11 & RP12, RP15 & RP16, and RP14 & RP6 (see below for details of primers and how gene targets were selected) that incorporated *Nde*I sites. For *in vitro* synthesis of dsRNA, the amplicons obtained above were cloned into pGEM®-T Easy (Promega, Southampton, UK) and transformed into *E. coli* JM109. Following selection of positive clones by ampicillin resistance, each insert was cloned into the *Eco*RI site of the *E. coli* T7 expression vector pLL10(20) with ampicillin selection of recombinant colonies. Plasmids pLLMW11 (with the *Vg* fragment), pLLMW14 (with the *NP2* fragment) and pLLMW15 (with the *NP1* fragment) were selected for *in vitro* synthesis of dsRNA in order to compare traditional microinjection methods with symbiont-mediated dsRNA delivery. The dsRNA was synthesized from these plasmids essentially as described by (8).

(3) Construction of expression vector to drive constitutive expression of dsRNA in *R. rhodnii*
In vitro synthesis of the expression cassette was outsourced to Eurofins MWG Operon (Ebersberg, Germany). This cassette comprised two convergent mutated copies of the promoter of the *Corynebacterium glutamicum dapA* gene, demonstrated to have good activity in *Rhodococcus erythropolis* (9), flanking a 245bp sequence of the *dagA* gene of *Streptomyces coelicolor*. Expression of a dsRNA copy of this sequence was designed as a negative control for subsequent knockdown experiments in *R. prolixus*. Recognition sites for specific restriction enzymes (*Eco*RI, *Nde*I, *Mlu*I, *Nhe*I and *Sal*I) were included at the ends and junctions of the promoter and *dag*A sequence to facilitate subsequent cloning steps. The cassette was introduced by Topo-TA cloning into the *E. coli* plasmid vector pCR2.1 (Invitrogen). Recombinant plasmids obtained were verified by restriction with *Eco*RI and named pMW101 (containing the recombinant dsRNA expression cassette; see Additional Methods). The cassette was subcloned into pIJ8600 as a *Bam*HI-*Eco*RV fragment to obtain pMW103 (pIJ8600 containing the expression cassette with *dagA* sequence). The *dagA* fragment itself was flanked by *Nde*I restriction sites to facilitate replacement by *R. prolixus* gene fragments. For RNAi experiments, the *dagA* sequence was replaced by a 342 bp fragment of *Vg* (pMW104), a 399 bp fragment of *NP2* (pMW105), and a 526 bp fragment of *NP1* (pMW106), obtained as described above as

amplicons from primers RP11 & RP12, RP15 & RP16, and RP14 & RP6.

(4) Construction of an RNaseIII deficient mutant of *R. rhodnii* (ME315)
Primers RR4 and RR5 were designed from ClustalW alignments of *rnaseIII* gene sequences from bacterial species related to *R. rhodnii*, and used to amplify a 300 bp product from *R. rhodnii.* To disrupt the gene, a kanamycin-resistance cassette flanked by *Mlu*I sites was generated by PCR using the template plasmid pME6 using primers RR17 and RR18. This amplicon was digested with *Mlu*I, ligated with pMW43 cut with *Mlu*I and cloned into *E. coli* JM109 with selection of both ampicillin and kanamycin. The resulting plasmid pMW44 was introduced into *R. rhodnii* by electroporation, and a mutant obtained after allelic exchange. Positive recombinants were selected for on TSA with kanamycin (50 µg/ml) and identified by Southern hybridization using a probe consisting of the *Sac*I fragment containing the *rnaseIII* gene isolated from pMW43.

(5) Construction of expression vector to drive constitutive expression of dsRNA in BFo2
A 138 bp synthetic expression cassette (Eurofins Genomics, Ebersberg, Germany), containing multiple restriction sites flanked by two copies of the modified constitutive promoter *Ptac* was used. The promoter sequences were designed to drive transcription in a convergent manner, to ensure transcription from both complementary strands of DNA fragments sub-cloned in the multi-cloning site. The synthetic expression cassette was cloned between the *Not*I sites of the ampicillin resistant plasmid pEX-A, generating plasmid pEX-A-Thrips cassette.
The methods used to help predict *F. occidentalis* gene fragments used as target candidates for dsRNA mediated interference were the same as for *R. prolixus* (see below). A 316 bp *F. occidentalis tubulin alpha-1 chain* gene fragment was chosen as the most suitable candidate template for dsRNA synthesis. The fragment was obtained by synthesis (Eurofins Genomics, Ebersberg, Germany), flanked by *Xba*I sites. This DNA fragment was provided as an insert cloned in the pEX-A vector.

The DNA fragments to be used as templates for dsRNA synthesis were sub-cloned into the *Xba*I site of pEX-A-Thrips cassette, and therefore flanked by convergent *Ptac* promoters to ensure constitutive transcription of two complementary RNA strands that would hybridise to generate the desired dsRNA. The resulting plasmid was pTub3. These constructs were verified by restriction and DNA sequencing.

(6) Generation of an RNaseIII deficient mutant of BFo2, BFo2α
The BFo2 *rnaseIII* gene was PCR amplified from wildtype BFo2 using primers RIIIBfo2F1 and RIIIBfo2R1 (see below). The 759 bp product was digested with *Eco*RI/*Hin*dIII and ligated into pIJ2925 previously digested at *Eco*RI/*Hin*dIII, generating plasmid pRNA1. Disruption of the *rnaseIII* gene in pRNA1 was achieved by EcoRV digestion and insertion of the apramycin resistance gene (flanked by T4 transcription terminators) excised as a *Hind*III fragment from plasmid pQM5062 and blunt-ended yielding pRNA2. To facilitate lambda-red mediated recombination, electrocompetent BFo2 cells were created as recommended for *E. coli* (10) and transformed by electroporation with plasmid pIJ790, creating strain BFo2/pIJ790. The disrupted *rnaseIII* gene was excised from pRNA2 by digesting with *Bgl*II and was introduced into BFo2/pIJ790 by electroporation. Transformants were selected by replica plating on selective media containing ampicillin or apramycin. Viable colonies were tested for the presence of the disruption by colony PCR using the primers BFo2RNasetestF and BFo2RNasetestR, permitting isolation of the *rnaseIII* mutant strain BFo2α. All plasmids were confirmed by restriction digestion and sequencing and the functionality of the BFo2 dsRNA expression system was confirmed as detailed below.

Selection of sequences for RNA interference

The chosen target sequences were assessed as having a low risk of potential cross-silencing. The online Deqor algorithm (11) was used to calculate all possible siRNA sequences from the

intended full dsRNA sequence, and evaluate them for cross-silencing potential by performing BLAST searches against the transcriptome of a selected insect species. Neither the *R. proxilus* nor WFT genomes were available during the dsRNA design stage, so the predicted transcriptomes of *Anopheles gambiae* and *Drosophila melanogaster* were both used. Two further online screens for potential cross-silencers were employed, which screened the *D. melanogaster* transcriptome: Snapdragon (http://www.flyrnai.org/cgi-bin/RNAi_find_primers.pl) and dsCheck (http://dscheck.rnai.jp/) (12). Finally, CLUSTAL W (http://www.ebi.ac.uk/Tools/msa/clustalw2/) was used to align each candidate dsRNA sequence with the full gene sequence for each of the other candidate genes for *R. prolixus*. This verified that none of the possible siRNAs had serious off-target sequence matches (i.e. >17 nucleotide perfect matches) that could lead to off-target effects in the candidate genes (13).

Persistence of recombinant *R. rhodnii* and BFo2

To determine whether recombinant *R. rhodnii* could successfully and reproducibly recolonize the gut of *R. prolixus* via feeding, and survive indefinitely in competition with the natural flora, *R. rhodnii*$_{eGFP}$ and dsRNA-expressing RNaseIII mutant *R. rhodnii* (ME315 strain) were grown in apramycin-supplemented TSB and membrane-fed to all developmental stages of *R. prolixus* as described above. Aposymbiotic insects were compared with non-sterile insects that had been reared from unsterilized eggs in the presence of the breeding colony. The presence or absence of recombinant *R. rhodnii* in the guts of *R. prolixus* was assayed using several methods. The midgut and hindgut of randomly-selected insects was dissected, surface-sterilized, homogenized and cultured on TSA plates with and without apramycin selection. The plates were incubated at 27°C in a humid environment for at least a week, preferably 10 days. EGFP-expressing apramycin-resistant *R. rhodnii* colonies were identified by morphology and fluorescence, counted and subjected to colony PCR to confirm their identity using *eGFP* primers (eGFP-f and eGFP-r; see below), and dsRNA-expressing *R. rhodnii* colonies were subjected to PCR using the relevant primers for the cloned *R. prolixus* gene fragments. The majority of insects, however, were retained for several months (being fed fortnightly thereafter with sterile horse blood). During this time, insects were moved to new cages at regular intervals, and the vacated cages were rinsed with TSB to dissolve feces. The presence or absence of recombinant *R. rhodnii* was assessed as above, with transformed bacteria usually being detectable in feces voided from 48h onwards after an infective feed.

To assess horizontal transfer, recipient non-sterile insects of various ages were introduced into sealed environments already containing donor insects of different ages carrying either eGFP-tagged *R. rhodnii* or the RNaseIII mutant ME315 expressing ds*dagA*. All the insects in the colony were given standard sterile blood feeds and after two such feeds, the introduced insects were removed, surface-sterilized and tested for the acquisition of eGFP-tagged or RNaseIII mutant *R. rhodnii*. This was confirmed by growth of CFUs on selective media from dissected gut contents, and colony-PCR using eGFP-specific primers where applicable. The data are separated by developmental stage of the insects to illustrate the comparative lack of transmission to 1st instar nymphs, which are significantly less able to acquire the symbiont than older (2nd instar stage upwards) insects (analysed by Fisher's exact test).

Recombinant BFo2 expressing *eGFP* were fed to thrips larvae using the artificial feeding system described above. Green fluorescence in the gut was observed through the intact cuticle of live insects anesthetized with CO_2 at 8h, and then at daily intervals during dietary exposure 7 days. The eGFP-expressing BFo2 were then withdrawn from the diet and the thrips continued to be monitored for the next 2-3 days.

Phenotypic assessment of *R. rhodnii*-mediated ds*NP1* and ds*NP2* knockdown in *R. prolixus*

Four groups of six 3rd instar *R. prolixus* were fed *R. rhodnii* ME315 expressing ds*NP1* or ds*NP2* during a blood meal, as described above. Further groups received a mixture of the two bacterial strains to assess a double knockdown phenotype, and groups of control insects were fed *R. rhodnii* ME315 expressing ds*dagA*. In parallel, *in vitro* synthesized dsRNA was micro-injected (2 µg/insect) for comparison. The insects were subsequently presented with sterile blood meals at 2 weekly intervals, which allows for the depletion of any pre-synthesized salivary components. After 8 weeks, the insects were sacrificed to allow examination of the salivary gland color and morphology.

Phenotypic assessment of *R. rhodnii*-mediated ds*Vg* knockdown in *R. prolixus*

The purpose of this knockdown experiment was to demonstrate a long-term, stable, symbiont-mediated RNAi phenotype on a large population of *R. prolixus*. Preliminary experiments demonstrated that a phenotype cannot be achieved if ds*Vg* if presented to the adult, rather than the 5th instar, developmental stage of the insects. Fifth instar male and female *R. prolixus* were therefore randomly assigned to experimental containers and fed *R. rhodnii* ME315 expressing either ds*Vg* or control ds*dagA* during a blood meal, as described above. A repeat blood feed, also containing recombinant bacteria, was given 2 weeks later. Insects that remained unfed were discarded. All subsequent meals were composed solely of sterile horse blood. At regular (approximately 10-day) intervals, a full population census was performed for every experimental group, to assess the RNAi effect on oogenesis and fecundity. The total number of live and dead insects of each developmental stage was recorded and, where possible, the sex. The number of hatched and unhatched eggs was also recorded, and dead insects and deformed adults females were removed during the census. The experiment was conducted for 200 days or until all the adults died, whichever occurred first. Because the experiment demanded large numbers of insects (168 insects exposed to ds*Vg*, and 163 exposed to ds*dagA*), repeat experiments were consecutively initiated in 17 staggered groups (each containing approximately 10 insects) over the course of a year. Adult males were always present in each cage to allow mating. Two groups of ds*Vg* treated insects and one ds*dagA* group were excluded from the experiment because no adults emerged in these containers.

Additional groups of insects, set up as above, were used for timed sacrifices to enable gene expression analysis by qRT-PCR. The gut contents of the sacrificed individuals were analyzed for the presence of recombinant *R. rhodnii*, as described above. Total RNA was isolated from individual insects (*R. prolixus*) harboring the recombinant bacteria using bash-beads and subsequently processed using the ZR Tissue and Insect RNA microprep kit (Zymo Research Europe GmbH, Freiburg, Germany). Genomic DNA was removed by on-column DNAse I digestion and the concentrations of RNA yields were normalised by diluting in nuclease free water. RNA integrity was determined by agarose gel electrophoresis, and for quantification and purity assessment a Nanodrop 2000 (ThermoScientific) was used. Reverse transcription was performed using the iScript Select cDNA synthesis kit (BioRad) with oligo dT primers to ensure only eukaryotic and not prokaryotic (symbiont) RNA was reverse transcribed. qRT-PCRs were performed in triplicate using SYBR Green Supermix (BioRad). Efficiency of qRT-PCR reactions was calculated using serial dilutions of pooled cDNAs, and specificity was assessed by melt-curve analysis. Relative gene of interest (GOI) transcript abundance was calculated by normalising to 18S rRNA. All qRT-PCR primers are listed below. In a further experiment, horizontal transmission of both the recombinant bacteria and their phenotype was assessed. Wildtype 5th instar insects were introduced to cages that had been previously occupied by insects in the above experiment. These cages thus contained the feces from insects that had been infected *per os* with recombinant bacteria. The fecundity of the new occupants was again assessed by population census, and persistence of the bacteria tested using gut and fecal samples as described above.

In all cases, the insects were periodically rehoused so that the vacated cages could be tested for the presence of excreted recombinant bacteria, using the method described above.

Phenotypic and molecular analysis of BFo2-mediated ds*Tub* knockdown in *F. occidentalis*

Sample *F. occidentalis* populations containing all developmental stages were orally infected with recombinant BFo2α expressing ds*dagA* RNA (control) and ds*Tub*, maintained as described above and monitored for a knockdown phenotype (mortality) after 4 days. An additional control was included which involved feeding of heat-killed BFo2α expressing ds*Tub*. To assess mRNA abundance, Juvenile thrips (1st and 2nd instar) were sampled 48 hours after infection with recombinant BFo2α, and levels of tubulin alpha1 mRNA quantified by qRT-PCR. Total RNA was isolated from pools of approximately 25 juvenile *F. occidentalis* homogenized with micro-pestles, and subsequently processed as described above. Relative gene of interest (GOI) transcript abundance was calculated by normalising to 18S rRNA.

Cucumber leaf damage assessment

Groups of three 15-day-old cucumber seedlings were each exposed to 50 larvae and 15 adult female *F. occidentalis* thrips that had been orally infected with BFo2α expressing ds*dagA* RNA (control), or ds*Tub* RNA (Tubulin KD). The experiments were performed under quarantine conditions inside ultra-fine mesh BugDorms (MegaView Science Co., Ltd., Taichung, Taiwan). After 5 days, digital images of the leaves were obtained to calculate the percentage of the leaf surface that was covered with lesions was assessed by Assess 2.0 image analysis software for plant disease quantification (14).

Statistical analyses

The *R. prolixus* population data (Table 1) were analyzed in SPSS (IBM Corp. Released 2013. IBM SPSS Statistics for Windows, Version 22.0. Armonk, NY: IBM Corp.). The analyses were conducted using a Generalized Linear Mixed Model (GZLMM), taking ‘treatment’ (factor) and ‘time period’ (covariate) as fixed effects and ‘insect container’ nested within ‘experiment number’ as a random effect. In all cases a scaled identity covariance structure provided the best fit when assessed using the Bayesian Information Criteria (BIC). We used the Binomial probability distributions to study death rates in adult females, adult males, offspring larvae, larvae molting and egg-hatching. When studying egg-laying and new hatchlings we instead used a Poisson distribution with the number of adult females taken as a fixed effect (covariate). The robustness of the results were verified by studying the impact of small changes to the model structure, none of which resulted in major changes to the findings.

The Mantel-Cox log-rank test was used to assess persistence of recombinant *R. rhodnii* in *R. prolixus*, and to assess the survival of *R. prolixus* after dsRNA injection. Fisher’s exact test (2-tailed) was used to examine developmental stage-specific changes in mortality rates of thrips infected with ds*Tub* or ds*dagA* expressing BFo2α. Unpaired 2-tailed t-tests compared relative transcript abundance values from qRT-PCR assays. Differences in cucumber leaf lesion coverage were analyzed by paired t-test (2-tailed).

References for Supplementary Methods:

1. Yanisch-Perron C, Vieira J, Messing J. Improved M13 phage cloning vectors and host strains: nucleotide sequences of the M13mp18 and pUC19 vectors. Gene. 1985;33:103–19.
2. Kieser, T., Bibb, M. J., Buttner, M. J., Chater, K. F., Hopwood DA. Practical Streptomyces Genetics. Norwich, UK: The John Innes Foundation; 2000. 1-613 p.
3. Gust B, Challis GL, Fowler K, Kieser T, Chater KF. PCR-targeted *Streptomyces* gene replacement identifies a protein domain needed for biosynthesis of the sesquiterpene soil odor geosmin. Proc Natl Acad Sci. 2003 Feb 18;100(4):1541–6.
4. Bishop, A., Fielding, S., Dyson, P. & Herron P. Systematic insertional mutagenesis of a streptomycete genome: A link between osmoadaptation and antibiotic production. Genome Res. 2004;14(5):893–900. doi:10.1101/gr.1710304
5. Sun J, Kelemen GH, Fernández-Abalos JM, Bibb MJ. Green fluorescent protein as a reporter for spatial and temporal gene expression in *Streptomyces coelicolor* A3(2). Microbiology. 1999;145(9):2221–7.
6. Blandin S, Moita LF, Kocher T, Wilm M, Kafatos FC, Levashina EA. Reverse genetics in the mosquito *Anopheles gambiae*: targeted disruption of the Defensin gene. EMBO Rep. 2002;3(9):852–6. doi: 10.1093/embo-reports/kvf180
7. Fernández-Martínez L, Bishop A, Parkes L, Del Sol R, Salerno P, Sevcikova B, et al. Osmoregulation in *Streptomyces coelicolor*: modulation of SigB activity by OsaC. Mol Microbiol. Blackwell Publishing Ltd; 2009 Mar 1;71(5):1250–62. doi:10.1111/j.1365-2958.2009.06599.x
8. Levashina EA, Moita LF, Blandin S, Vriend G, Lagueux M, Kafatos FC. Conserved role of a complement-like protein in phagocytosis revealed by dsRNA knockout in cultured cells of the mosquito, *Anopheles gambiae*. Cell. 2001;104(5):709–18.
9. Knoppová M, Phensaijai M, Veselý M, Zemanová M, Nešvera J, Pátek M. Plasmid vectors for testing *in vivo* promoter activities in *Corynebacterium glutamicum* and *Rhodococcus erythropolis*. Curr Microbiol. Springer New York; 2007;55(3):234–9.
10. Sambrook JF, Russell DW. Molecular Cloning: A Laboratory Manual (3rd edition). Cold Spring Harbor Laboratory Press; 2001. 2100 p.
11. Henschel A, Buchholz F, Habermann B. DEQOR: a web-based tool for the design and quality control of siRNAs. Nucleic Acids Res. 2004;32(suppl_2):W113–20.
12. Naito Y, Yamada T, Matsumiya T, Ui-Tei K, Saigo K, Morishita S. dsCheck: highly sensitive off-target search software for double-stranded RNA-mediated RNA interference. Nucleic Acids Res. Oxford University Press; 2005 Jul 1;33(Web Server issue):W589–91.
13. Kulkarni MM, Booker M, Silver SJ, Friedman A, Hong P, Perrimon N, et al. Evidence of off-target effects associated with long dsRNAs in *Drosophila melanogaster* cell-based assays. Nat Meth. 2006 Oct;3(10):833–8.
14. Lamari L. Assess 2.0. Image Analysis Software for Plant Disease Quantification. American Phytopathological Society. American Phytopathological Society Press; 2008.

Oligonucleotides used for PCR and qRT-PCR:

**Oligonucleotides**	
eGFP-f	AAGTTCAGCGTGTCCGGC
eGFP-r	TTCTTCTGCTTGTCGGCCA
RP11	GTTTCTCATATGAGCCACAACTTTCGACAACA
RP12	GTTTCTCATATGGCATAAACATGCACCAGAA
RP15	GTTTCTCATATGCAGTGGGTGTAAGTGGAA
RP16	GTTTCTCATATGGTGGAAATGAAGTCGCTGAA
RP14	GTTTCTCATATGGAGTCAGTGGAGACTGT
RP6	CCGCATCTTTTTGGTGAGTT
RR4	CACCGCTCGTAYGCGTA
RR5	TGGAGTCGATTCTCGGCG
RR17	agaacgcgtAAGCGAACCGGAATTGCCAG
RR18	caaacgcgtGAATACTCATACTCTTCCTT
RIIIBfo2F1	TTAGAATTCGTTGATACAGCCCTGTTTCATGTGC
RIIIBfo2R1	TTTAAGCTTTTAGTCAGTGCTTGCTCTGCAGC
BFo2RNasetestf	AGCGGATACCGTAGAAGCAC
BFo2RNasetestR	TAGGCCGGTAACGGTAAGTG
**Oligonucleotides for qRT-PCR**	
RP18SQPCRF	GTTGGTATTGATGTACGCTGGA
RP18SQPCRR	CCTACGGAAACCTTGTTACGA
VgQPCRF1	GCTGTCTTCACCTTGTAT
VgQPCRR1	CGTCCATCTATTAACTACTCA
FoQPCR18SF	GAAGGATTGACAGATTGA
FoQPCR18SR	TAGAGTCTCGTTCGTTAT
FoQPCRTubUpF	TTGAAGAAGCATCCTAAC
FoQPCRTubUpR	TTGAGAAGTAGTTGAGATTA

Gene targets used to generate synthetic DNA fragments to be used as templates for dsRNA synthesis:

Target gene (Genbank)	Plasmid name	Length of synthetic sequence
F. occidentalis tubulin alpha-1 chain (GT305545.1)	pEX-A-tubulin alpha-1	316 bp
R. prolixus vitellogenin (GAHY01001271.1)	pMW104	342 bp
R. prolixus nitrophorin-2 (U70582.1)	pMW105	399 bp
R. prolixus nitrophorin-1 (L39654.1)	pMW106	526 bp
S. coelicolor dagA (control) (X05811.1)	pMW103 / pEX-A-*dagA*	245 bp

Recombinant cassette in pMW101:

```
  1 ATGTGGCACC TACAAGGGTC GCCAGGTCAC CGCAGCGATC TGATTCGTCG GTTTCCGGTG
 61 AACACCGACT CTCGCAGCCA CCCCGAGCGC GGCGACGAGG GCCATGAGCC CCTCCTCGCC
121 GCGCTCGGCG TTACGCTCGA TCCCGAACTG CTGACGCTCG CGCTCACCCA CCGCTCGTAC
181 GCGTACGAGC ACGGTGGGCT ACCCACCAAC GAACGCCTCG AGTTTCTCGG CGACTCGGTG
241 CTGGGCCTCG CCGTGACCGA GCGGCTCTAT CTCGATCACC CCACTCGCCC CGAGGGCGAA
301 CTCGCGAAGA TCCGCGCCAG CATCGTCAAC ATGCACGCGC TCGCCGAGGT CGCGCGGGAG
361 CTGGGCCCCG GTGGGCTCGG TGCCCACATC CTGCTCGGCA AGGGCGAGGA GATGACGGGC
421 GGCCGCGACA AGCCGAGCAT CCTCGCCGAC GGCATGGAGT CGCTCCTCGG CGCGATCCAC
481 CTGCAGCACG GCATCGACGT CGCGCGCGGC GTCGTGTCGC GGCTCTTCGG CGCCCTCCTC
541 GAACGTGCGC CCGGACTCGG CGCCGGCCTC GACTGGAAGA CAAGCCTGCA GGAGCTGACG
601 GCCGAACGCG GCCTCGGCGT CCCCGCGTAC GAGATCACCG CGACCGGGCC CGATCACGAC
661 AAGGAGTTCA CCGCGACCGT CGTCGTCGCG GGTGGCGGAC TCGGCGTCGG CGTCGGACGC
721 ACGAAGAAGG AAGCCGAGCA GAAGGCCGCC GCGACCGCGT GGACCGAGCT GAGCGGCGGC
781 CCCGCCGACG TCGCCGGCGC GTAG
```

Nucleotide sequences 1-6 and 463-468 are recognition sites for *Eco*RI; nucleotide sequences 7-102 and 360-456 are the sequences of the two copies of the convergent modified *dapA* promoter; nucleotide sequences 103-108 and 355-360 are recognition sites for *Nde*I; nucleotide sequence 109-348 is the 5' end of the *dagA* gene of *Streptomyces coelicolor*; nucleotide sequences 349-354 is a recognition site for *Mlu*I; nucleotide sequences 457-462 is a recognition site for *Sal*I.

Plasmid map of pMW103 and schematic showing principle of symbiont-mediated RNAi in *R. prolixus*.

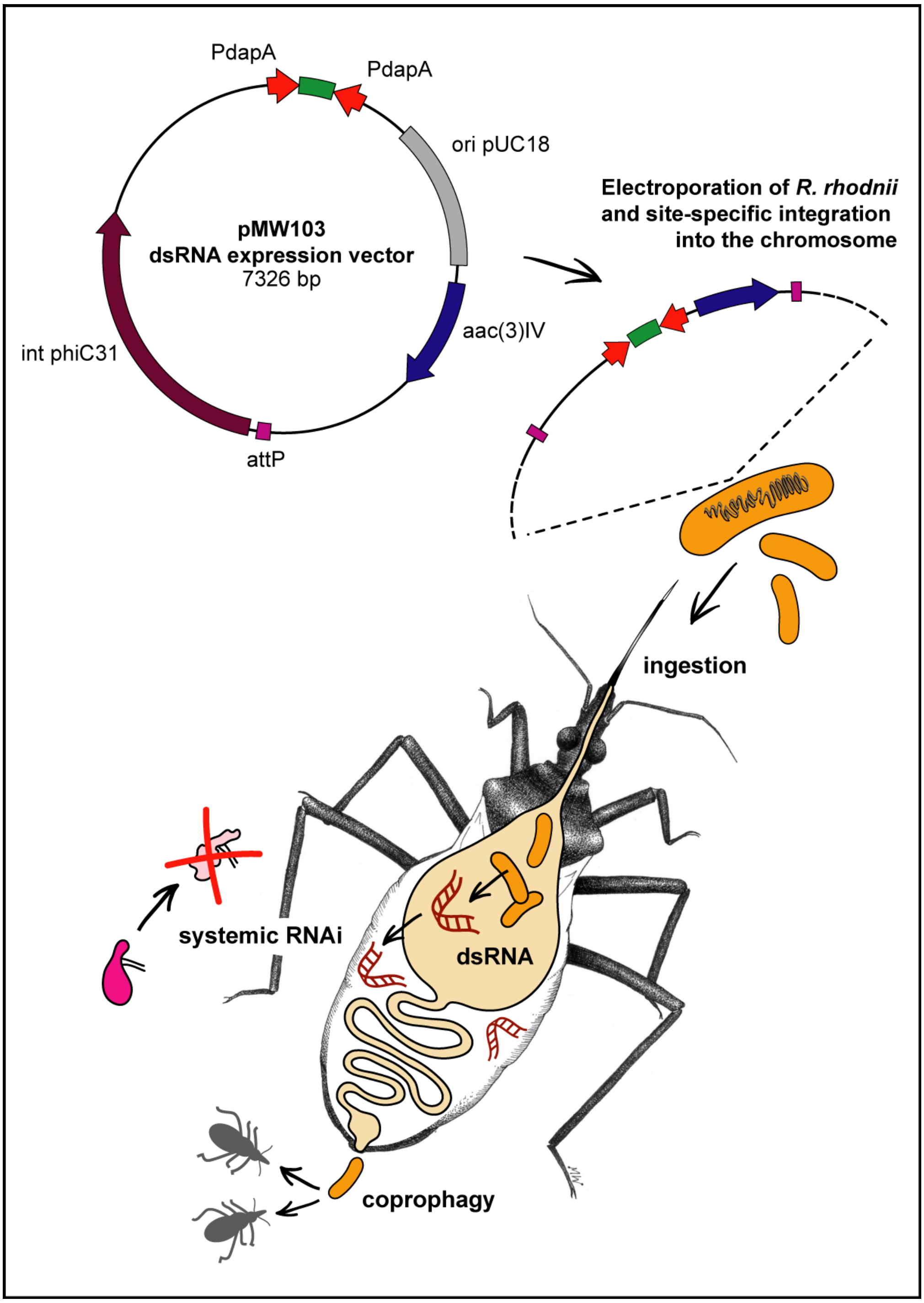

Thrips cassette:

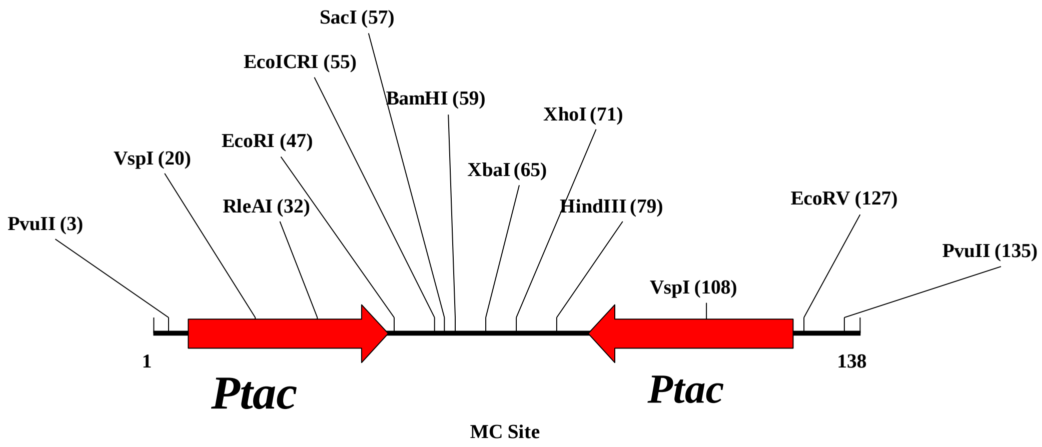

Converging promoters are indicated in red. Notice that *Ptac* promoters do not contain an operator sequence, therefore are not subject to LacI repression.

Map of pEX-A-Thrips cassette:

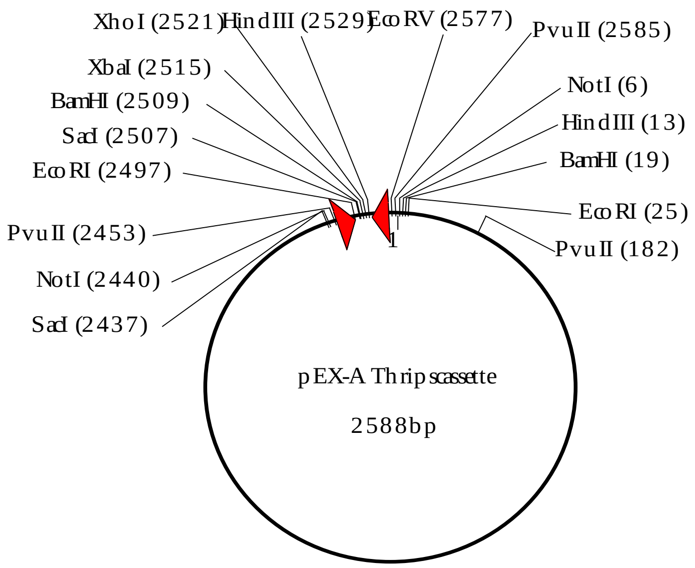

Plasmid maps of pElong1 (a) and pTub3 (b)

Red arrows indicate *Ptac* promoters and the blue section indicates the position of the dsRNA template sequence.

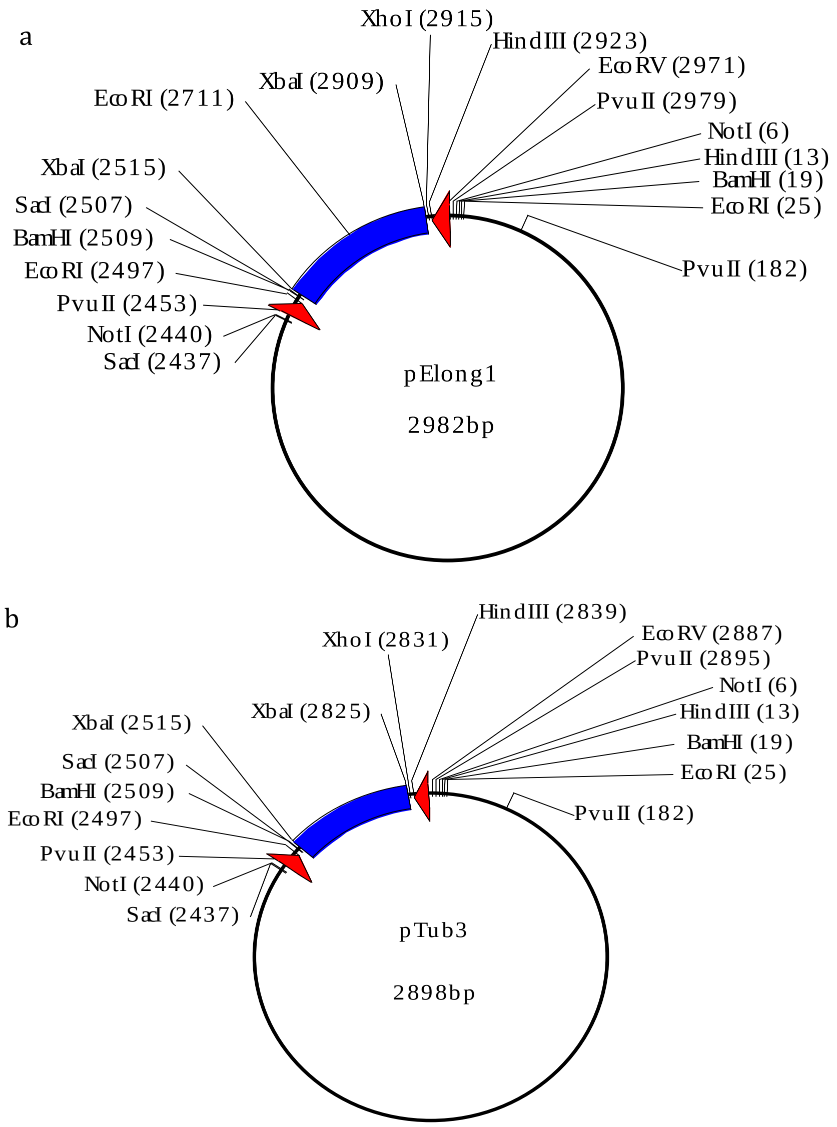

Functionality of BFo2 dsRNA expression system

Cell pellets of BFo2α containing the dsRNA expression plasmids were mixed with equal volumes of RNA Protect (Qiagen Ltd., Manchester, UK), and total RNA was isolated using the RNeasy mini RNA isolation kit (Qiagen Ltd.). After removal of genomic DNA by on-column DNase I treatment (Qiagen Ltd.), strand-specific reverse transcription was performed using the iScript Select reverse transcription kit (BioRad) including the gene-specific primers and primer enhancer (GSP). Strand-specific real-time amplification was performed in triplicate using SYBR-Green Supermix (BioRad). No-template controls were used to assess the extent of genomic DNA carry-over.

SUPPLEMENTARY FIGURES

SUPPLEMENTARY FIGURE 1

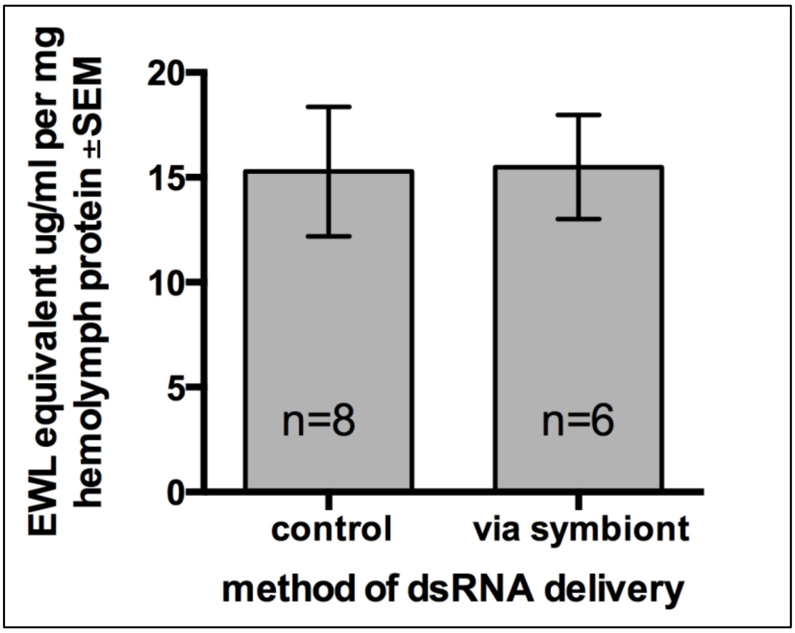

Hemolymph (blood) lysozyme activity in *R. prolixus* 48h following symbiont-mediated dsRNA delivery.
Insects were either fed with ds*dagA*-expressing *R. rhodnii* ME315 (RNaseIII mutant strain symbionts), or left unchallenged (controls). Lysozyme activity is expressed as the equivalent activity for a known concentration of commercially-available egg white lysozyme (EWL). No significant increases in lysozyme activity were detected above basal levels (paired t-test, 2-tailed). Similar results were obtained for hemolymph phenoloxidase activity.

SUPPLEMENTARY FIGURE 2

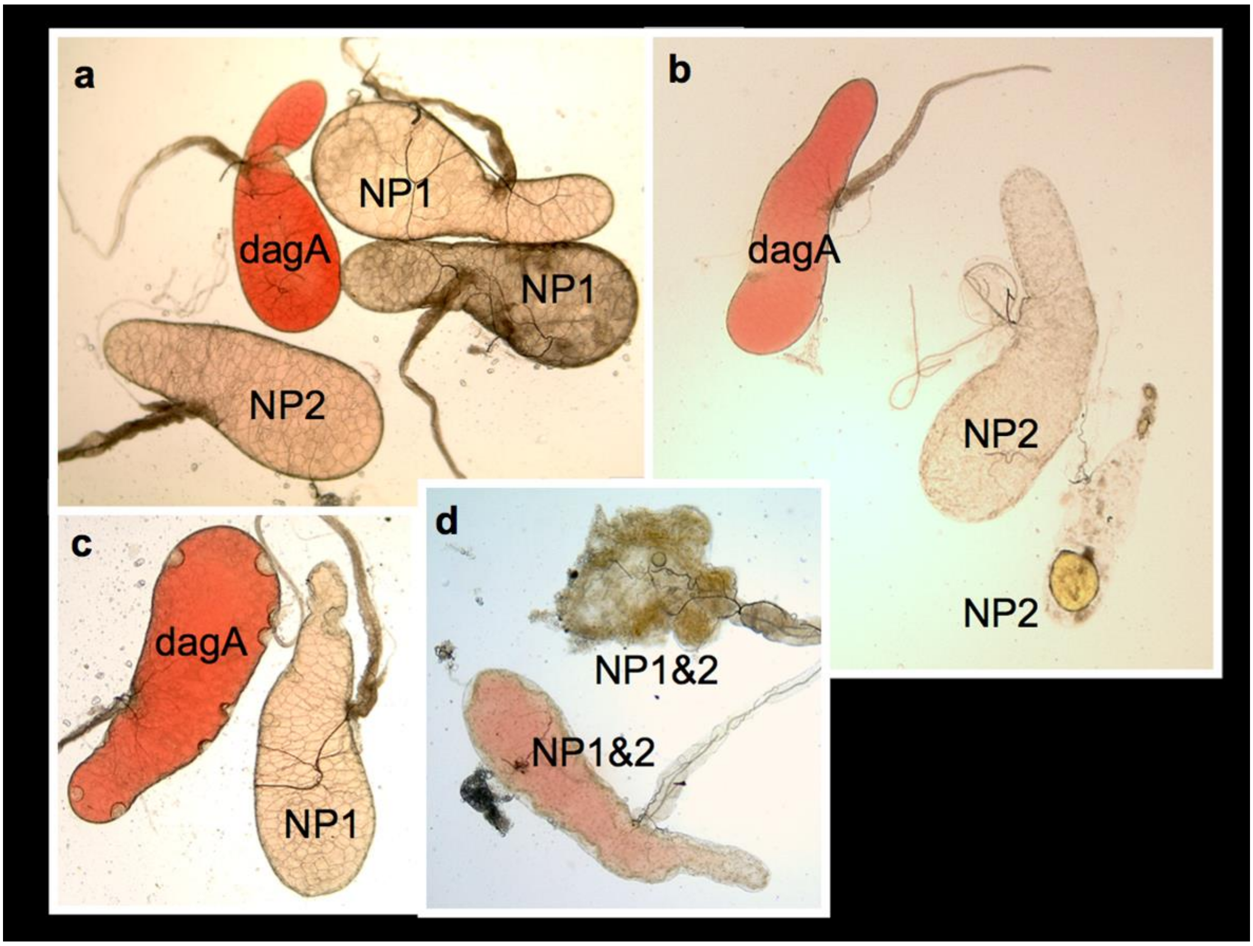

Salivary glands from *R. prolixus* 3rd instars exhibiting Nitrophorin-1 (NP1) and Nitrophorin-2 (NP2) knockdown phenotypes. Salivary glands from knockdown groups show a dramatic change in colouration from cherry-red to pale, compared with glands from ds*dagA* (control) treated insects. (a) Phenotype following injection of dsRNA (2 µg / insect) for comparison. (b-d) Phenotypes 8 weeks after *per os* infection with *R. rhodnii* ME315 (RNaseIII deficient mutant) expressing dsRNA: (b = ds*NP2*, c = double *NP1/NP2* knockdown, d = ds*NP1*).

SUPPLEMENTARY FIGURE 3

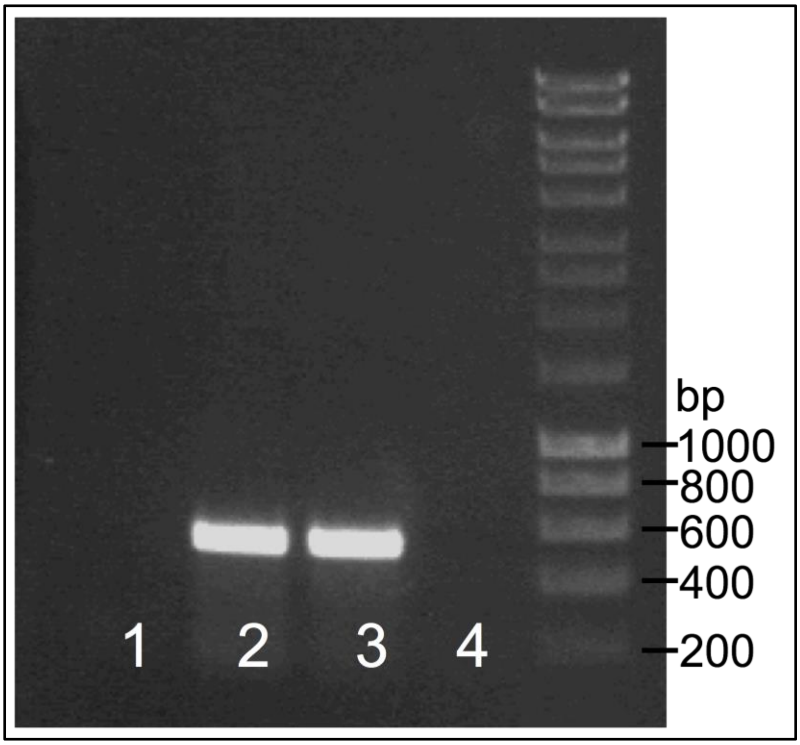

Constitutive expression of dsRNA by RNaseIII deficient mutant *R. rhodnii*. Total RNA was extracted from *R. rhodnii* cultures either as a non-mutant or RNaseIII mutant form (strain ME315). The RNA was subjected to 2-step RT-PCR using gene-specific primers for the dsRNA fragment, and visualized on a 1% agarose gel (the example presented is for *dsNP1* expression). Lane 1: recombinant *R. rhodnii* containing the *dsNP1* expression cassette but with functional RNaseIII (initial template RNA concentration 252 ng/ul). Lane 2: recombinant RNaseIII deficient mutant *R. rhodnii* expressing *dsNP*1 (initial template RNA concentration 220 ng/ul). Lane 3: *in vitro* synthesized *dsNP1* as a positive control (initial template RNA concentration 30 ng/ul). Lane 4: no-template control.

SUPPLEMENTARY FIGURE 4

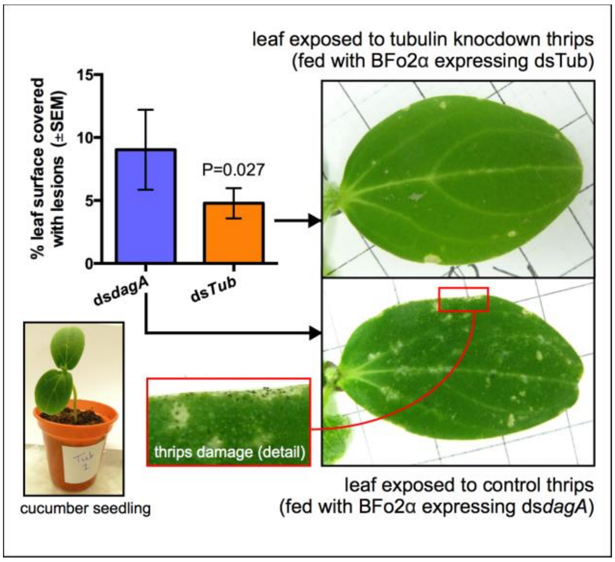

RNAi of *Tubulin* expression in *F. occidentalis*: control of leaf damage.
In this pilot study, groups of three 15-day-old cucumber seedlings were each exposed to 50 larvae and 15 adult female *F. occidentalis* that had been orally infected with bacteria (BFo2) expressing ds*dagA* (control), or ds*Tub*. Size-normalized % leaf surface covered by lesions was assessed five days later and was significantly lower in plants exposed to the *Tubulin* knockdown insects (paired t-test, 2-tailed).
